# A Context-Aware Navigation Framework for Ground Robots in Horticultural Environments

**DOI:** 10.3390/s24113663

**Published:** 2024-06-05

**Authors:** Peiqi Jin, Tongxiang Li, Yaoqiang Pan, Kewei Hu, Nuo Xu, Wei Ying, Yangwen Jin, Hanwen Kang

**Affiliations:** 1 College of Engineering, South China Agriculture University, Guangzhou 510070, China; 20213142022@stu.scau.edu.cn (Y.P.); huck_weeeee@whu.edu.cn (K.H.); 20233175014@stu.scau.edu.cn (N.X.); yangtsejin@stu.scau.edu.cn (Y.J.); hanwen.kang@outlook.com (H.K.); 2 TongTai Technology Co., Ltd., Qingdao 266000, China

**Keywords:** horticulture, context-aware, ground robots, navigation framework, path planning

## Abstract

Environmental mapping and robot navigation are the basis for realizing robot automation in modern agricultural production. This study proposes a new autonomous mapping and navigation method for gardening scene robots. First, a new LiDAR slam-based semantic mapping algorithm is proposed to enable the robots to analyze structural information from point cloud images and generate roadmaps from them. Secondly, a general robot navigation framework is proposed to enable the robot to generate the shortest global path according to the road map, and consider the local terrain information to find the optimal local path to achieve safe and efficient trajectory tracking; this method is equipped in apple orchards. The LiDAR was evaluated on a differential drive robotic platform. Experimental results show that this method can effectively process orchard environmental information. Compared with vnf and pointnet++, the semantic information extraction efficiency and time are greatly improved. The map feature extraction time can be reduced to 0.1681 s, and its MIoU is 0.812. The resulting global path planning achieved a 100% success rate, with an average run time of 4ms. At the same time, the local path planning algorithm can effectively generate safe and smooth trajectories to execute the global path, with an average running time of 36 ms.

## 1. Introduction

**Figure 1:**
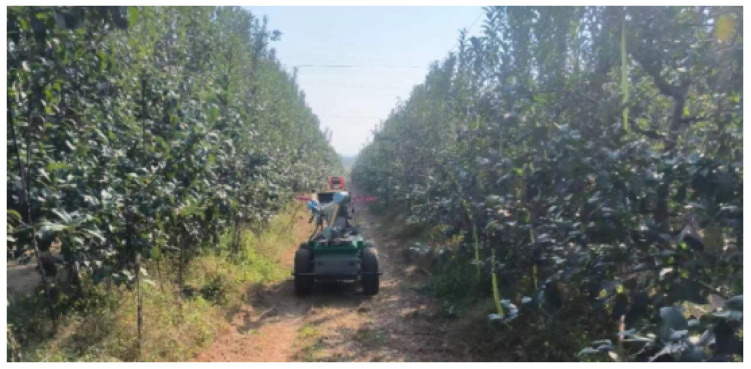
Mobile robot in orchard.

Robots with autonomous exploration capabilities have become a key direction in precision agriculture research. When intelligent robots navigate autonomously in orchards, they usually use a series of sensors and technologies to perceive and understand the surrounding environment, in order to effectively plan and execute navigation tasks [1]. Autonomous navigation [2] gives robots the ability to perform tasks independently in complex and dynamic environments, and includes five steps: environment perception, positioning, map construction, path planning and motion control. Map construction aims to model the orchard environment so that the robot can fully understand the operating environment.As shown in Figure 1, the robot performs tasks in the orchard.Path planning plans the robot’s movement on the map to complete the corresponding. In short, the key to a robot’s autonomy lies in its ability to perceive the environment, understand the environment, make decisions based on the information in the environment, and perform the corresponding actions.

Simultaneous localization and mapping (SLAM) [3] uses mapping, positioning, and pose estimation algorithms to build a map. The robot starts to move from an unknown position in an unknown environment. During the movement, it positions itself according to the position and the map, and at the same time, based on its own positioning, builds incremental maps to realize autonomous positioning and navigation. The system uses sensors such as cameras, light detectors, ranging (LiDAR), radar and IMU to obtain visual and motion information from the surrounding environment [4]. This information is used on the front end of SLAM to move the robot’s movement mileage and construct the surrounding environment. In the SLAM backend, the environment map is fine-tuned through loop closure detection and overall map optimization. Ultimately, an accurate landmark or point cloud map can be obtained through these processes. Semantic SLAM technology based on deep learning can construct dense point cloud maps containing semantic information. Compared to sparse point cloud maps, semantic point cloud maps contain more sufficient information, which helps robots fully understand the actual scene content and improves the map’s reliability and readability. Semantic SLAM can avoid complex imperative interactions and extract implicit semantic instructions from human action instructions. For example, in an orchard [5], if the robot knows where the target fruit tree is, it can automatically find the path to the given location and complete the job without a human tapping the screen every time to tell the robot exactly where to go. At the same time, fruit identification and positioning under dynamic conditions is also an important task, because it can significantly improve the harvesting efficiency [6]. In this work, by taking advantage of LiDAR-based mapping methods, a novel two-step semantic mapping framework that can achieve robotic autonomy in orchards is proposed. Firstly, we present a novel 3D detection method to accurately identify and localize objects on point cloud maps directly. Secondly, we develop a mapping framework to construct visibility graph-map for robot motion planning. Specifically, details of our contributions are as below.

- On the basis of the original point cloud, a detection network is created that can quickly and accurately identify the three-dimensional environment point cloud. By processing the original three-dimensional point cloud, different categories of instance-level information are obtained.
- A new semantic mapping framework is developed through semantic processing of point cloud information and extraction and analysis of environmental terrain through deep learning networks.
- We apply the semantic mapping and navigation framework on mobile robots and conduct experiments in apple orchards to verify the effect.

The rest of this paper is organized as follows. Section 2 surveys related work. An overview of the system-aware and semantic mapping framework in Section 3 describes the composition of the point cloud network detection framework. Section 3.5.3 introduces the architecture and implementation of the semantic mapping framework. Section 5 introduces the sensor composition and work flow of the experimental robot, as well as specific experimental results, and then the conclusions are given in Section 6.

## 2. Related Works

### 2.1. Robot Navigation in Horticulture

Semantic information [7] plays a crucial role in SLAM systems. This is specifically reflected in the environment modeling, positioning and navigation of robots, as well as improving the robustness and accuracy of robot environment perception by fusing semantic information with sensor data [8]. A multi-sensor-based navigation solution is proposed to enable the robot to perform effective path planning and navigation by integrating landmarks and obstacle information that need to be identified in the environment into a semantic map. Cheng Peng et al. [9] proposed an end detection method for robots to realize autonomous navigation and obstacle avoidance in orchards without GNSS signals, which used the drastic changes in the statistical distribution of points sensed by the depth camera at the end of the robot, based on the real-time perception and response of the robot to the surrounding environment, combined with the semantic map, and further improved the navigation accuracy and safety of the robot in the orchard. In response to the needs of orchard spraying operations, Zhou, Xinzhao et al. [10] constructed an environmental perception and map construction strategy based on three-dimensional LiDAR in the complex environment of the orchard, based on the three modules of “perception–decision–control” of the unmanned system, and implemented it under different working conditions. The obstacle avoidance performance and navigation accuracy of the autonomous navigation spray vehicle were verified, and the feasibility of the developed system was verified. Li, Zhiqiang et al. [11] aimed at the problem of loss of navigation information obtained by plant protection robots under traditional visual navigation methods. They proposed using LiDAR point cloud data to supplement machine vision, and based on the characteristics of the actual environment, use point cloud data to identify obstacles between rows and columns of objects, obtain auxiliary navigation information, and provide guarantees for the stable operation of robots in colleges and universities. Denis F. Wolf et al. [12] combined robot learning techniques with standard mapping algorithms to develop maps that represent environments and navigability. At this stage, robots capable of precise environmental recognition, robust and flexible motion, and accurate plant modeling remain an area for exploration.

### 2.2. Review of Robot Mapping

Semantic information plays a crucial role in advancing concurrent robotic SLAM systems. SLAM++ [13] introduces an SLAM technology that uses RGB-D data for object perception, which is achieved by jointly optimizing the position of the camera and the object. This approach relies on matching the objects identified by the 3D object detector with a pre-scanned object database for graph optimization, enabling the construction of maps at the object semantic level. Sucar et al. [14] combined a monocular SLAM algorithm based on a Kalman filter with object detection technology to estimate the global scale of the 3D reconstruction model. This research method integrates object detection and SLAM technology, and avoids the need to rely on a database of pre-scanned objects as prior information. However, this approach fails to generate maps that contain rich semantic information. Traditional simultaneous localization and mapping (SLAM) methods cannot extract semantic information from the scene or meet the high-level task requirements of robots, and the construction efficiency of 3D maps is low. In order to solve this problem, Lei Lai et al. [15] proposed a three-dimensional semantic graph construction system to construct a three-dimensional semantic graph. First, the current position of the camera is estimated and optimized based on the ORB-SLAM algorithm to obtain a globally consistent trajectory and posture; then, a semantic segmentation network is designed to predict the semantic category of each pixel. Combining the semantic information with the object point clouds generates three-dimensional semantic point cloud information. Lin et al. [16] created a semantic map by modeling environmental objects using voxels and cuboids, and used the semantic information matching of objects for closed-loop correction. This method not only constructs a semantic map, but also improves the positioning accuracy of the SLAM system by using semantic information. Zhong et al. [17] designed a robot vision system that combines SLAM with object detection based on deep neural networks to filter out the interference of unreliable features of dynamic objects, and construct an object-level semantic map with good real-time performance. Despite these advances, these methods are not explicitly designed for agricultural and horticultural contexts, which hinders the adaptability and reliability of robotic automation in these environments.

**Figure 2:**
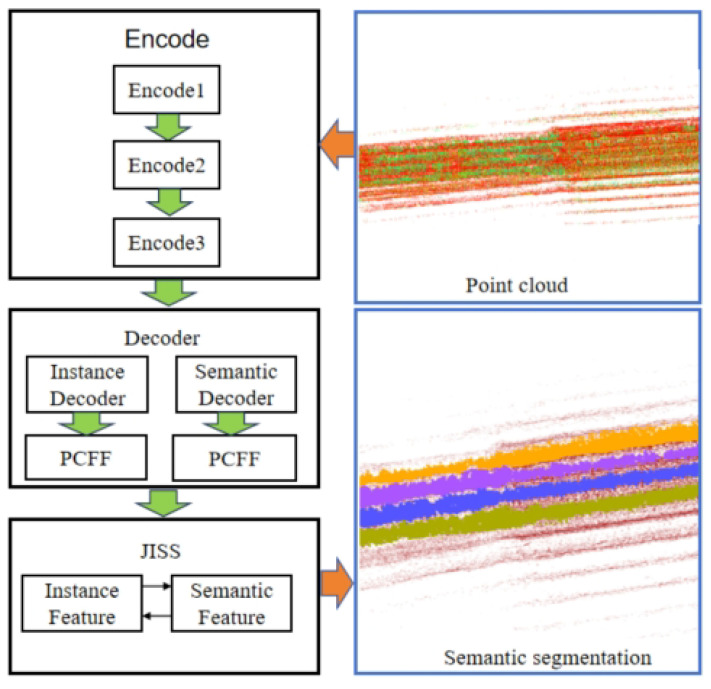
Demonstration of semantic map results.

At present, great progress has been made in object detection in 2D by extracting semantic information, but object detection based on a 3D point cloud is still a challenging subject. The three-dimensional point cloud obtained by SLAM method provides a more comprehensive representation of the object distribution scene. The perception and semantic mapping framework proposed in this article is shown in Figure 2. The framework consists of three modules: mapping, perception and semantic processing. It uses autonomous mobile robots as carriers, and is equipped with LiDAR to collect orchard data. The mapping process uses the LIO-SAM [18] algorithm to process the collected data. This algorithm combines IMU and LiDAR data to achieve real-time estimation of the robot’s position and attitude in the environment. On this basis, the information in the map is used to detect and locate fruit trees in the orchard on the network model proposed by us. Finally, the semantic processing module extracts terrain map information through a Cloth Simulation Filter, and combines the extracted point cloud map topology information with the network model prediction results to construct a semantic map. The robot can clearly understand the distribution of fruit trees through semantic maps and use this information for autonomous path planning in the orchard [19].

## 3. Methods

### 3.1. Context-Aware Mapping

The network structure of the perception model designed in this article for three-dimensional point clouds is shown in Figure 2. It includes a shared feature encoder, two parallel branch decoders, and the decoder includes a feature fusion module and a joint segmentation module. There are two parallel branch decoders: one is for extracting semantic features from raw point clouds in orchards, the other is used for semantic segmentation tasks. Finally, the JISS module obtains and processes semantic features and instance features, and outputs two feature matrices. One of the shaped matrices is used to predict semantic categories. Another shape is an instance feature matrix that is used to predict the instance label for each point. In the embedding space, the clustering algorithm (DBSCAN) [20] is used to divide the identified points according to their different densities to form clusters with a reachable density, thereby identifying the natural clustering structure in the data. To put it simply, the points belonging to the same instance object are very close, while the points of different instances are far away from each other, thereby better dividing the environmental semantic information. The point cloud clustering algorithm is shown in Algorithm 1.
**Algorithm 1:** DBSCAN
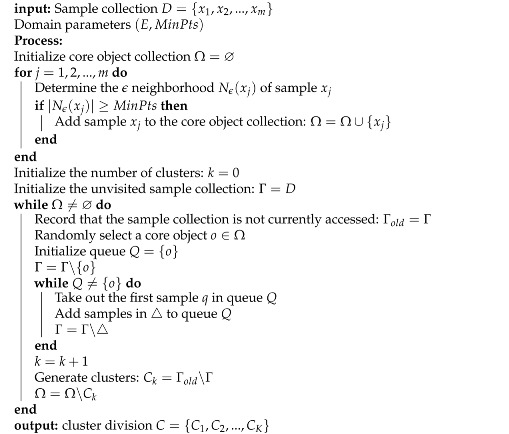


### 3.2. Geometric Mapping

**IMU preintegration factor:** At time *t*, the measured values of the original acceleration ${\hat{a}}_{t}$ and angular velocity ${\hat{\omega }}_{t}$ of the inertial measurement unit are defined as follows:

(1) $$\begin{array}{cc} \omega & =\hat{\omega }-b_{\omega }-n_{\omega }\\ a & =R_{\mathrm{WB}}\left(\hat{a}-b_{a}-n_{a}\right)-g \end{array}$$

where $\hat{\omega }$ and $\hat{a}$ are the measurement value of IMU in the body coordinate system at time *t*, $b_{\omega }$ is the angular velocity bias, and $b_{a}$ is the acceleration bias. $R_{\mathrm{WB}}$ is the rotation matrix from body coordinate to world coordinates, g is the constant gravity vector in the world coordinates. From this, we can obtain the formulas for the rotation, position and speed from time *t* to time t+Δt:

(2) $$R_{t+\Delta t}=R_{t}\mathrm{Exp}(\omega_{t}\Delta t)$$

(3) $$\begin{array}{cc} p_{t+\Delta t} & =p_{t}+v_{t}\Delta t+\frac{1}{2}g\Delta t^{2}+\frac{1}{2}R_{t}a_{t}\Delta t^{2} \end{array}$$

(4) $$v_{t+\Delta t}=v_{t}+g\Delta t+R_{t}a_{t}\Delta t$$

According to Equations (2)–(4), we can obtain the the calculation equation of the IMU preintegration factor:

(5) $$\Delta v_{ij}=R_{i}^{T}\left(v_{j}-v_{i}-g\Delta t_{ij}\right)$$

(6) $$\Delta p_{ij}=R_{i}^{T}\left(p_{j}-p_{i}-v_{i}\Delta t_{ij}-\frac{1}{2}g\Delta t_{ij}^{2}\right)$$

(7) $$\Delta R_{ij}=R_{i}^{⊺}R_{j}$$

**LiDAR odometry factor:** In LIO-SAM, the edge features and plane features are extracted for each frame of radar data frame, and $F_{i}^{e}$ and $F_{i}^{p}$ are used to represent the edge features and plane features in the data frame i and form the current radar. The composition of data frame $F_{i}$, $F_{i}$ is as follows:

(8) $$F_{i}=\left\{F_{i}^{e},F_{i}^{p}\right\}$$

Convert the associated *n* sub-keyframes and the set of recent frames $\left\{F_{i-n},\cdots ,F_{i}\right\}$ to the world coordinate system, and merge them into a voxel map $M_{i}$, where the transformation relationship between each frame and the world coordinate system is $\left\{T_{i-n},\cdots ,T_{i}\right\}$, and $M_{i}$ consists of two types of voxel maps, one of which is an edge feature voxel map $M_{i}^{e}$ that consists of edge features FieW in the world coordinate system, and the other is the surface feature voxel map $M_{i}^{p}$, which consists of edge features FipW in the world coordinate system.

(9) $$M_{i}=\left\{M_{i}^{e},M_{i}^{p}\right\}$$

(10) Mie=FieW∪Fi−1eW∪…∪Fi−neW

(11) Mip=FipW∪Fi−1pW∪…∪Fi−npW

In LIO-SAM, the edge features and surface features are extracted from the radar point cloud of the new frame, and the initial transformation from the mobile robot’s base coordinate system to the world coordinate system is calculated through IMU pre-integration ${\tilde{T}}_{i+1}$, thereby converting the features in the radar point cloud frame to the world coordinate system to obtain {Fi+1eW,Fi+1pW}, and the corresponding edge features and plane features in the map are found in the corresponding voxel.

(12) $$F_{i+1}=\left\{\begin{array}{c} F_{i+1}^{e},F_{i+1}^{p} \end{array}\right\}$$

When the corresponding feature is found for each edge and planar feature in the radar frame matching, the distance between the feature and its edge or planar counterpart is calculated using Equations (13) and (14), respectively.

(13) $$d_{e_{k}}=\frac{\left|\left(p_{i+1,k}^{e}-p_{i,u}^{e}\right)\times \left(p_{i+1,k}^{e}-p_{i,v}^{e}\right)\right|}{\left|p_{i,u}^{e}-p_{i,v}^{e}\right|}$$

(14) $$d_{p_{k}}=\frac{\left|\begin{array}{c} \left(p_{i+1,k}^{p}-p_{i,u}^{p}\right)\\ \left(p_{i,u}^{p}-p_{i,v}^{p}\right)\times \left(p_{i,u}^{p}-p_{i,w}^{p}\right) \end{array}\right|}{\left|\begin{array}{c} \left(p_{i,u}^{p}-p_{i,v}^{p}\right)\times \left(p_{i,u}^{p}-p_{i,w}^{p}\right) \end{array}\right|}$$

where *k*, *u*, *v*, *w* are the index numbers corresponding to the features, $p_{i+1,k}^{e}$ is a single edge feature in the Fi+1eW feature, and $p_{i,u}^{e}$ and $p_{i,v}^{e}$ are the two points in $M_{i}^{e}$ that form the straight line where the edge is located. Correspondingly, $p_{i+1,k}^{p}$ is a planar feature in the surface feature Fi+1pW, and $p_{i+1,u}^{p}$, $p_{i+1,v}^{p}$ and $p_{i+1,w}^{p}$ are the three points that make up the surface feature in $M_{i}^{p}$.

Solve the optimized transformation by minimizing Equation (15) using the Gauss–Newton method. Finally, the transformation relationship between two radar data frames (that is, the radar odometry factor) is obtained through Equation (16).

(15) $$\min_{T_{i+1}}\left\{\sum_{p_{i+1,k}^{e}\in^{\prime }F_{i+1}^{e}}d_{e_{k}}+\sum_{p_{i+1,k}^{p}\in^{\prime }F_{i+1}^{p}}d_{p_{k}}\right\}$$

(16) $$\Delta T_{i,i+1}=T_{i}^{T}T_{i+1}$$

### 3.3. Context-Aware Perception

**Figure 3:**
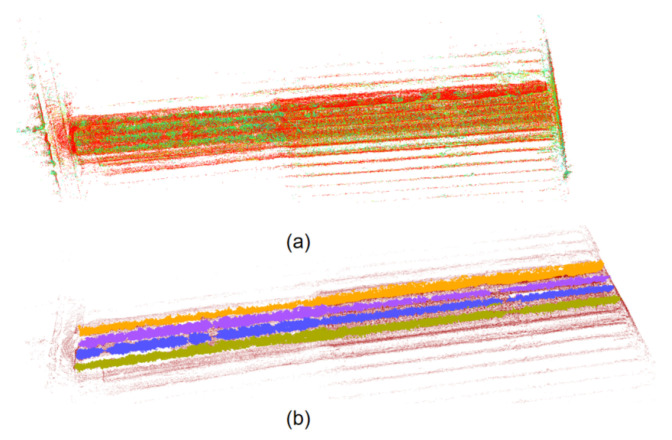
Mapping and semantic mapping results of orchards: (**a**) colorized map, (**b**) semantic map of orchards.

Orchard environmental surveying and mapping is performed based on LIO-SAM, which proposes a framework for tightly coupled LiDAR inertial navigation odometry utilizing GT-SAM. It can realize high-precision, real-time trajectory estimation and mapping of mobile robots. In the experiments of this article, RS Helios 32-line LiDAR and external IMU (frequency 400 hz) are used as the data source for LIO-SAM mapping. According to the previous flight performance in orchards using GPS as the data source for drone positioning, the presence of many obstacles in orchards will seriously affect the GPS signal and affect the flight stability of drones. So, we use IMU measurements instead of GPS when performing LIO-SAM mapping to infer the robot’s movement. The estimation based on IMU pre-integration corrects the point cloud information detected in the environment, and provides a preliminary estimable solution for LiDAR odometry optimization. On this basis, LiDAR will estimate the positioning deviation generated by the IMU through range measurement. With this complete solution, LIO-SAM can effectively simulate and describe the orchard’s geometric features. As shown in Figure 3, we use camera data to color the orchard to clarify the characteristics of the fruit trees. Figure 3a is the distribution of the orchard. Figure 3b is the orchard semantic map extracted using the method of this article. Through the fruit tree semantics’ map distribution, we can see that the global map of the orchard is very unstructured, and at the same time, the distribution of the extracted path points in the map is very uneven. We effectively simulate and describe the orchard geometric characteristics.

### 3.4. Roadmap Generation

**Figure 4:**
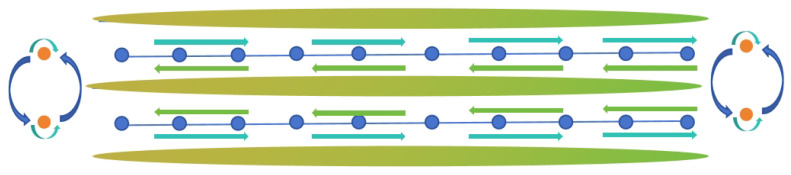
Orchard visibility map path planning results.

During actual robot travel, the robot faces complex challenges when performing a U-turn between trees, and often requires human intervention to successfully complete the turn. To overcome this problem, we impose one-way travel restrictions on each path. For each tree, we specify two access points with different directions on both sides of the tree, and connect nodes with the same direction to the surroundings of the tree, forming a loop. The entire orchard is divided into three rows, each with two paths. By leveraging the semantic information, we successfully transformed the originally scattered and unordered orchard environment into a structured graph mapping with nodes and edges. After completing the mapping, the system can automatically build a navigation map of the orchard to provide reliable navigation support for the robot. Planning renderings are shown in Figure 4.

### 3.5. Navigation System

#### 3.5.1. Global Path Generation

The autonomous navigation robot built in this article uses the Dijkstra [21] algorithm to calculate the shortest path from the starting point to the end point in the global path planning of the orchard, so that the robot can complete relevant tasks in the shortest possible time. The relevant definitions are as Algorithm 2:
**Algorithm 2:** Algorithm for Dijkstra
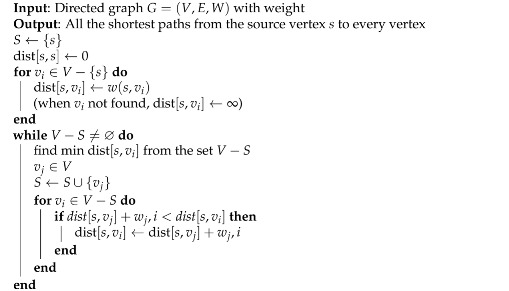

1. At the beginning of the algorithm, the selected source points are put into the set *S*.
2. The shortest path from *s* to itself without self-loop is 0.
3. When the vertex $v_{i}$ is not in the set *S*, it starts to enter the loop.
4. Assign the weight between the source point *s* and the point $v_{i}$ to $\mathrm{dist}[s,v_{i}]$. Since it is a directed graph, when the source point *s* does not point to any other vertex outside the set *S*, dist[s,u]=∞. It can be understood that starting from the source point *s* at this time, $v_{i}$ cannot be reached for the time being. However, as the set *S* expands later, all vertices can be reached from the source point *s*. At this point, the first for loop ends.
5. If the set V−S is not the empty set, enter the loop.
6. Select the vertex *v* with the shortest distance in the shortest path relative to the set *S* in the set V−S after the first for loop.
7. Merge this vertex *u* into the set *S* to achieve the purpose of expanding the set *S*.
8. Merging vertex $v_{i}$ into set *S* may have an impact on the length of the shortest path of other vertices relative to set *S*, so enter the inner for loop to update the affected ones.
9. That is, if the length of the shortest path from the source point *s* to the vertex $v_{j}$ selected in step 6 relative to the set *S*, plus the distance between vertex $v_{j}$ and vertex $v_{i}$, denoted as $w_{i,j}$, is less than the length of the shortest path from the source point *s* to vertex $v_{i}$ relative to the set *S*.
10. Then, update the shortest path from the source point *s* to vertex $v_{i}$ relative to the set *S* to be the shortest path from the source point *s* to the vertex $v_{j}$ selected in step 6 relative to the set *S*, plus the weight $w_{i,j}$ of the edge from vertex $v_{j}$ to vertex $v_{i}$.

#### 3.5.2. Local Motion Optimization

Using the global path planning module based on Dijkstra’s algorithm, the shortest path is determined for the mobile robot from the current position to the target position on the semantic topology map. However, due to the unpredictability and dynamic changes in the actual environment, global path planning may produce sharp path turns, so local path planning becomes an indispensable strategy [22]. This article uses TEB-planner as the local planner. This algorithm is an efficient path-planning algorithm. It is specially designed for dynamic environments, and can provide real-time obstacle avoidance solutions for mobile robots. The algorithm is unique in its use of temporal elastic bands (TEB), a technique that optimizes paths in time and space, allowing mobile robots to navigate flexibly and efficiently in complex environments. Central to the design of TEB-planner is its concept of constraint functions, which are responsible for introducing various restrictions in the path planning process to ensure that the generated path is not only safe and feasible, but also meets specific operating efficiency and smoothness requirements. These constraint functions mainly include obstacle avoidance constraints, path running time constraints, and path smoothness constraints. Obstacle avoidance constraints ensure that obstacles are avoided during the path planning process and provide a safe navigation path; the goal of the fastest path constraints is to enable the mobile robot to complete its task in the shortest time; the path smoothness constraints ensure that the mobile robot can complete its tasks in the shortest time, avoiding too sharp steering or acceleration changes. Through the comprehensive consideration and optimization of these constraint functions, TEB-planner can achieve fast, efficient, and safe path planning in dynamic environments.

#### 3.5.3. System Architecture

**Figure 5:**
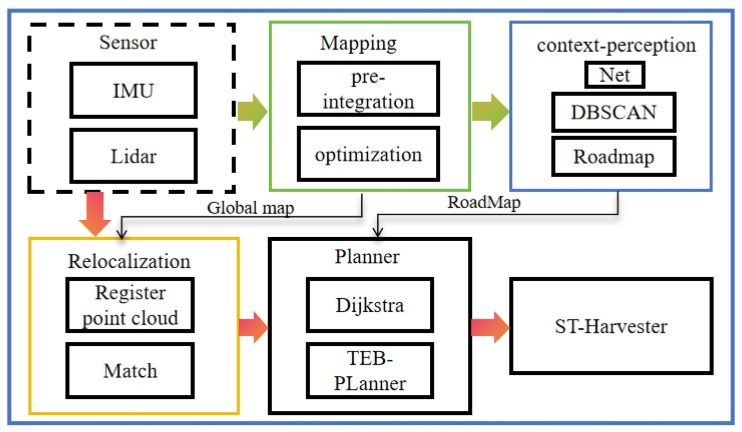
Software architecture of system.

The mobile robot system architecture constructed in this study is shown in the Figure 5 respectively. The core of the system is an industrial computer, which runs a Robot Operating System (ROS). Through the driver provided by the ROS, the system can obtain sensor data. First, the LIO-SAM mapping module was used to conduct global three-dimensional point cloud mapping of the orchard. After the mapping was completed, the feature encoder designed in this study extracted the feature information of the point cloud map in the perception module and generated an information feature map, and passed this instance information to the semantic topology mapping module. This module further refined the ground and obstacle information in the orchard map based on semantic information, and extracted structured information and accessible nodes through a straight line detection algorithm (Hough line transformation) based on the spatial distribution of fruit trees. Finally, the semantic topology mapping module combines semantic data and node information to create a semantic topology map containing directed edges. The global planner in the navigation module performed global path planning based on this map, and determined a series of global path points. Subsequently, the local planner performed local path planning based on these path points to ensure that the path satisfied obstacle avoidance, motion feasibility, and optimal path constraints.

#### 3.5.4. Relocalization

The normal distributions transform (NDT) was initially proposed by Biber for LiDAR scan registration [23], and this technique was subsequently adapted for 3D applications [24].

The principle of the NDT algorithm is to first use the reference point cloud to construct a normal distribution of the multi-dimensional variables. When the point cloud to be registered is transformed and matches the reference point cloud to a high degree, the probability density of the transformed point cloud will be very high. The transformation matrix that maximizes the probability density of the source point cloud can be obtained through optimization.

Each point in the point cloud to be registered can obtain a probability value on the normal distribution model of the reference point cloud. This probability value represents the possibility that the point belongs to the distribution within the corresponding voxel. The probability representation model is Equation (17).

(17) $$p\left(x\right)∼exp\left(-\frac{{\left(x-\mu \right)}^{T}\Sigma^{-1}\left(x-\mu \right)}{2}\right)$$

where *x* represents points in the point cloud, μ represents the mean of these points, Σ represents the covariance matrix of these points.

The mean is calculated as:

(18) $$\mu =\frac{1}{n}\sum_{i}^{n}x_{i}$$

The covariance matrix is calculated as:

(19) $$\Sigma =\frac{1}{n}\sum_{i}^{n}\left(x_{i}-\mu \right){\left(x_{i}-\mu \right)}^{T}$$

The current LiDAR scan can be represented by point cloud $X=x_{1},x_{2},\cdot ,x_{n}$, and the probability density function of these points is p(X). Assuming that there is a transformation function $T(\vec{p},x)$, the point $x_{i}$ in the current frame coordinate system can be mapped to the world coordinate system through its pose $\vec{p}$.

The optimal pose $\vec{p}$ should minimize the following likelihood function:

(20) $$-\log\Psi =-\sum_{k=1}^{n}\log\left(p(T\left(\vec{p},x_{k}\right))\right)$$

In the 3D NDT algorithm, the transformation function using Euler angles is:

(21) $$\begin{array}{cc} T_{E}\left({\vec{p}}_{6},x\right) & =R_{x}R_{y}R_{z}x+t\\ \; & =\left[\begin{array}{ccc} c_{y}c_{z} & -c_{y}s_{z} & s_{y}\\ c_{x}s_{z}+s_{x}s_{y}c_{z} & c_{x}c_{z}-s_{x}s_{y}s_{z} & -s_{x}c_{y}\\ s_{x}s_{z}-c_{x}s_{y}c_{z} & c_{x}s_{y}s_{z}+s_{x}c_{z} & c_{x}c_{y} \end{array}\right]x\\ & +\left[\begin{array}{c} t_{x}\\ t_{y}\\ t_{z} \end{array}\right] \end{array}$$

where ${\vec{p}}_{6}={\left[t_{x},t_{y},t_{z},\phi_{x},\phi_{y},\phi_{z}\right]}^{T}$, $R_{x}$, $R_{y}$, and $R_{z}$ represent the rotation matrix around the x, y, and z axes, respectively, $c_{i}=\cos\phi_{i}$ and $s_{i}=\sin\phi_{i}$, i=x,y,z.

The Jacobian matrix of the variables ${\vec{p}}_{6}$ in Equation (21) is:

(22) $$\frac{\delta T_{E}\left({\vec{p}}_{6},\vec{x}\right)}{\delta \vec{p}}=\left[\begin{array}{cccccc} 1 & 0 & 0 & 0 & c & f\\ 0 & 1 & 0 & a & d & g\\ 0 & 0 & 1 & b & e & h \end{array}\right]$$

where

(23) $$\begin{array}{cc} & a=x_{1}\left(-s_{x}s_{z}+c_{x}s_{y}c_{z}\right)+x_{2}\left(-s_{x}c_{z}-c_{x}s_{y}s_{z}\right)+x_{3}\left(-c_{x}c_{y}\right),\\ & b=x_{1}\left(c_{x}s_{z}+s_{x}s_{y}c_{z}\right)+x_{2}\left(-s_{x}s_{y}s_{z}+c_{x}c_{z}\right)+x_{3}\left(-s_{x}c_{y}\right),\\ & c=x_{1}\left(-s_{y}c_{z}\right)+x_{2}\left(s_{y}s_{z}\right)+x_{3}\left(c_{y}\right),\\ & d=x_{1}\left(s_{x}c_{y}c_{z}\right)+x_{2}\left(-s_{x}c_{y}s_{z}\right)+x_{3}\left(s_{x}s_{y}\right),\\ & e=x_{1}\left(-c_{x}c_{y}c_{z}\right)+x_{2}\left(c_{x}c_{y}s_{z}\right)+x_{3}\left(-c_{x}s_{y}\right),\\ & f=x_{1}\left(-c_{y}s_{z}\right)+x_{2}\left(-c_{y}c_{z}\right),\\ & g=x_{1}\left(c_{x}c_{z}-s_{x}s_{y}s_{z}\right)+x_{2}\left(-c_{x}s_{z}-s_{x}s_{y}c_{z}\right),\\ & h=x_{1}\left(s_{x}c_{z}+c_{x}s_{y}s_{z}\right)+x_{2}\left(c_{x}s_{y}c_{z}-s_{x}s_{z}\right). \end{array}$$

## 4. Global Relocation Experiment

**Figure 6:**
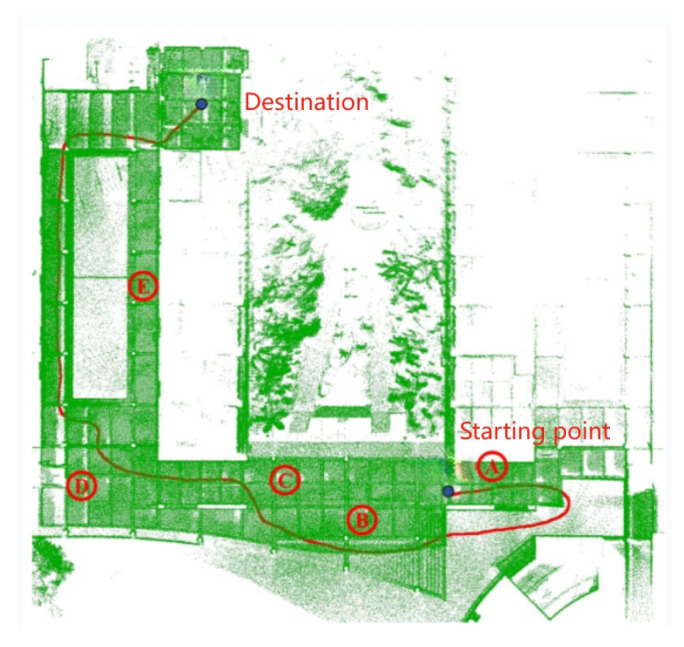
Relocation trajectory comparison.

The experimental location of this section is on the campus of the South China Agricultural University. The visualization result of the trajectory at the relocation starting point A is shown in Figure 6. At this time, the average positioning error is 8.8 cm and the maximum positioning error is 120 cm; the maximum positioning error occurs at the starting point. From the relocation trajectory (Figure 6), we can see that the change in trajectory of A is larger at the starting point, because the NDT matching result at the beginning has not completely converged, and it has been in a state of matching the local map and the global map. After walking a certain distance, the matching results become better, the relocation accuracy becomes higher, and the trajectory is closer to the real trajectory.

In order to verify the accuracy of the NDT algorithm, this section of the experiment selected multiple locations in the school as starting points, as shown in Figure 6 from A to E. The experiment was repeated ten times. The results of the relocation experiment based on the NDT algorithm are shown in Table 1.

The NDT-based relocation algorithm has a 100% success rate of relocation at A and C, 90% at B and D, and 70% at E, and the average positioning accuracy is significantly higher than that of the other locations. The main reason is that the space at E is narrow, there are too many repeated scenes, and there are fewer features, while the space changes at A, B, C, and D are obvious, there are more features, and the success rate and accuracy of retargeting are high.

## 5. Experiment and Discussions

### 5.1. Runtime Module

**Figure 7:**
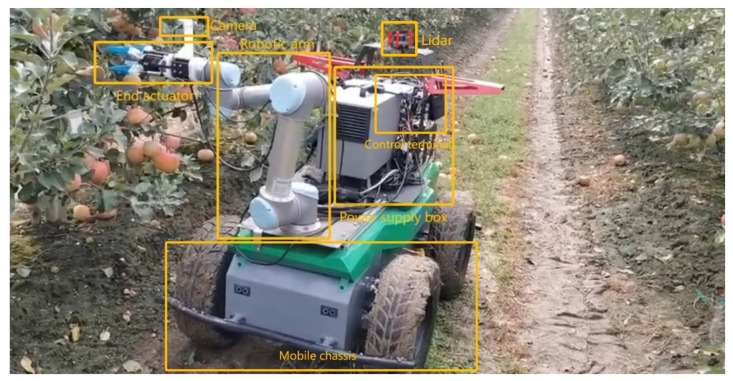
Movable robot structure.

The structure of the movable robot is shown in Figure 7. The robot is equipped with a 32-line LiDAR and an external nine-axis IMU. The LiDAR acquisition frequency is 20 Hz, and the data acquisition frequency of the external IMU is 400 Hz. Data collected through these sensors can be used to create an initial point cloud map of the orchard using mapping algorithms. In this specific experiment, the LIO-SAM algorithm run by the robot uses RS-Helios and external IMU data to construct an orchard point cloud image in XYZI format. The realsense-D435 depth camera is used to acquire color images and combine these images without affecting the structure of the generated map. For visualization of color maps, in Ubuntu 20.04, the sensor suite on the robot is connected to the central computer (NVIDIA Xavier) via the Robot Operating System (ROS Noetic). Data from the RS-Helios is transferred to a central computer via an Ethernet port, and using the rsLiDAR-ros driver, the ROS system passes the sensor data from the sensor suite to the mapping module. The mapping module generates a global point cloud map. The perception module proposed in this article perceives object-level fruit tree information from the map and generates semantically rich maps through semantic mapping. Finally, through the ROS system, the constructed semantic map information and navigation information are visualized, and interact with the operator.

To evaluate the applicability of the proposed semantic topology map and navigation framework for mobile robot phenotyping tasks in orchards, this paper conducts field tests in an irregular outdoor orchard. By comparing the performance differences between the global navigation framework, based on semantic topology maps in this paper, and the traditional TEB planner in actual operations, the ST-100 differential drive robot is used to navigate along the global path points determined in the traversable topology map. Based on the characteristics of the differential drive robot, the traversable topology map was appropriately adjusted, and the two key points for adjusting the steering were strategically placed in the last tree row so that the mobile robot could change direction or switch channels as needed. Considering that the orientation of the destination point is critical to the phenotype collection task, a predetermined orientation was specially set for each target location. In order to comprehensively evaluate its performance, the global navigation module of this study was compared with the native TEB-planner and the TEB-planner with manually optimized obstacle maps. We demonstrate the necessity of the navigation framework proposed in this article in mobile robots performing tasks.

### 5.2. Evaluation in Simulation Environments

We conducted three different sets of experiments to compare the practical application differences of the proposed semantic extraction framework compared with the two other feature extraction methods. Through comparisons of the specific experimental data, it can be seen that the semantic feature extraction framework proposed in this article has a much shorter extraction time for the same environment map than the two other methods, with a specific time of 0.1681 s. And, through a comparison of the semantic segmentation index (MIoU), the specific data are 0.812. It can better reflect the efficiency of the proposed feature extraction framework (Table 2).

In the orchard, we conducted three different sets of experiments to verify the effectiveness of the proposed global navigation network-based framework.

**Figure 8:**
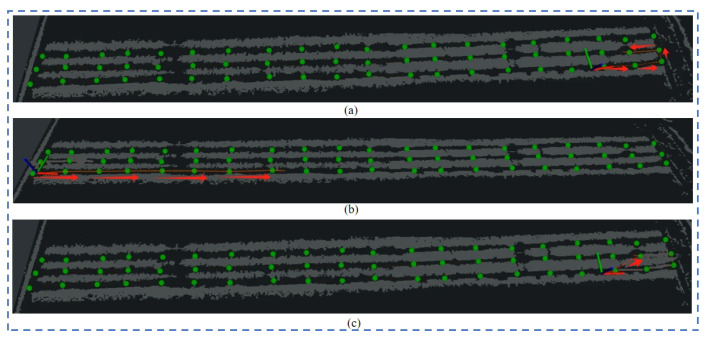
Experiment 1: the target point is in the same direction as the robot’s movement.

Experiment 1: The direction of travel of the robot to the target point set in the experiment is the same as the direction of the shortest path planned by the robot. The visualization results of the proposed planning algorithm are as shown in Figure 8a–c. As shown, all can reach the target point according to the planned shortest path. The planned path of the native TEB-planner planner is as shown in Figure 8c. The planned path will pass through the crop rows to reach the target point. It does not reach the target point in the specified direction, so it fails. Based on the method proposed in this article, the navigation framework and the TEB-planner planning path using the optimized map can successfully reach the target point according to the set direction.

**Figure 9:**
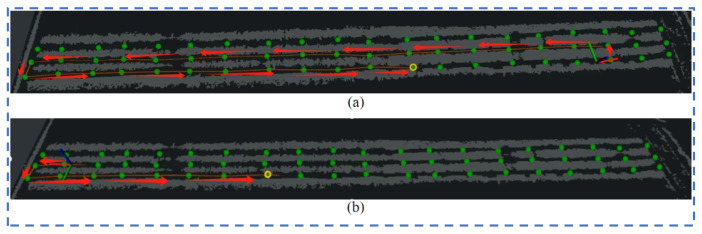
Experiment 2: the target point is opposite to the movement of the robot.

Experiment 2: The direction of the robot’s distance from the target point set on the map is opposite to the robot’s traveling direction. The native TEB-planner, the TEB-planner based on the optimized map, and the test plan under the proposed navigation framework are also used. The visualization effects are shown in Figure 9a,b. Neither the TEB-planner algorithm nor the map-based optimization results can reach the target point according to the planned shortest path, while the visibility map adjusts the vehicle direction by moving an extra distance to ultimately achieve the navigation task.

**Figure 10:**
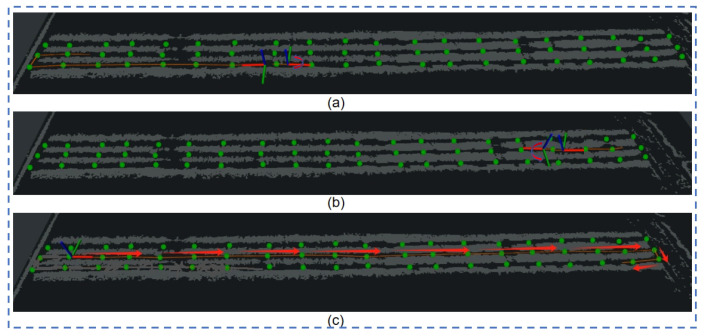
Raw TEB-planner path planning results (1), (2); visibility graph path planning results (3).

Experiment 3: When the set target point is close to the robot, but opposite to the direction of the robot’s travel, the visualization results are shown in Figure 10a–c. Regardless of whether it is based on the native TEB-planner, or uses the map-optimized TEB-planner, both planning methods will rotate at the appropriate position and travel to the target location, but planning in this way is not advisable in an orchard. In contrast, the visibility-based semantic map approach guides the robot to open space at both ends of each channel, and the robot repositions itself and reaches the predetermined goal in the desired direction.

TEB-planner uses global waypoints in the visibility graph as the local planner. The route map takes into account both the vehicle’s current direction and the direction it will take when it reaches each destination, ensuring the operational direction of the orchard navigation. In comparison, the original TEB planner does not consider the vehicle direction at the end of the global path, which may lead to the problem of opposite directions. In addition, we randomly selected 10 target points on the visibility map of each scene, compared them with the TEB planner, and recorded the planning time (see Table 3). Results show that the visibility map is significantly faster to compute, making it the top planner with a minimal performance penalty. Through these three different preset target point types, and the use of different path planning strategies, we can conclude through comparison that the autonomous driving robot based on the visibility map method can meet the requirements of path planning in the phenotype acquisition task.

### 5.3. Demonstration in Applications

**Figure 11:**
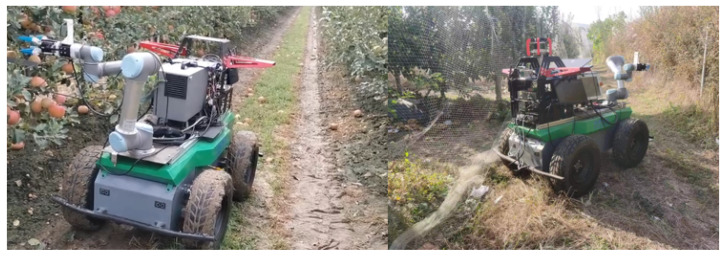
Field demonstration in the wild apple orchard.

**Figure 12:**
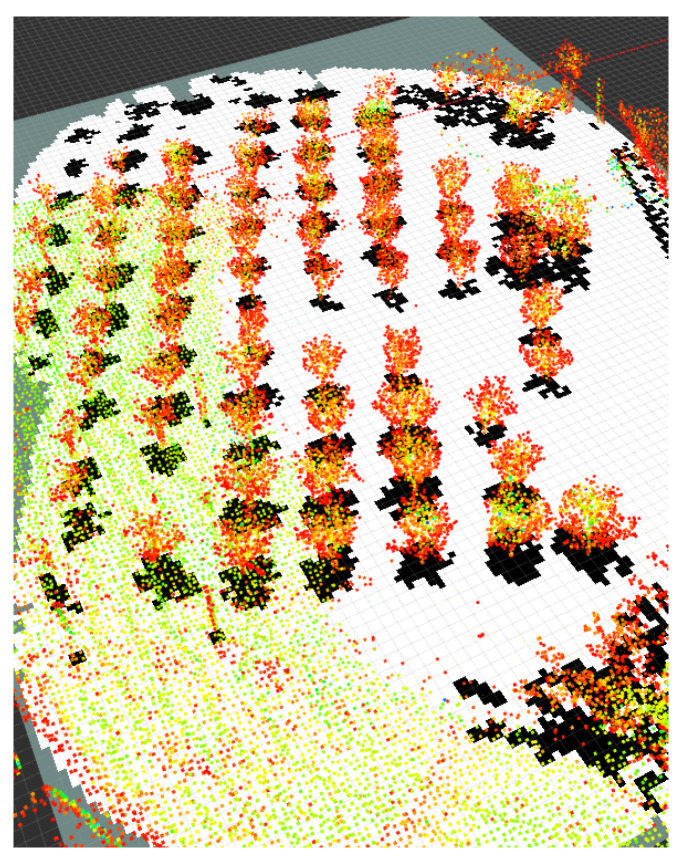
Orchard point cloud map.

**Figure 13:**
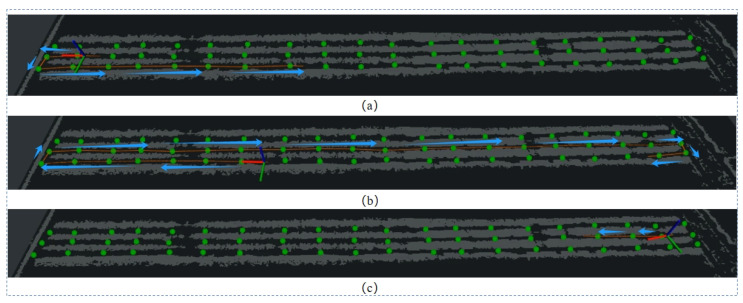
Visibility graph path planning results in apple orchard.

We tested the semantic mapping and navigation framework in an apple orchard, following the network framework proposed in this paper, using it as an upper-level planner and TEB-planner as a local planner using global paths in the visibility graph. In terms of the proposed path map, the current driving direction of the vehicle is considered in conjunction with reality, as well as the actual direction of the vehicle when traveling and reaching the target point, ensuring the specific operating direction of the vehicle in the orchard navigation. From the actual topographic map of the orchard in Figure 11, and the point cloud of the orchard in Figure 12, it can be seen that the overall layout of the fruit trees in the apple orchard is irregularly distributed. So, it is not suitable for the perception framework we proposed. In order to solve this problem, we artificially set relevant nodes on the visualization graph to represent the location of the fruit trees, and set U-turn points at both ends of the tree in the visibility graph. The path planning results are shown in Figure 13a,b. As shown in Figure 13c, it can be seen from the visualization results that, based on the artificially given nodes, the framework proposed in this article can still provide reasonable and effective path planning effects for autonomous driving robots in the orchard.

### 5.4. Discussion

In this study, we developed an orchard robot platform based on deep learning and autonomous navigation. Sensors such as LiDAR are used to sense and map orchard data. Through the proposed point cloud feature recognition and extraction network, various point cloud information in the environment is extracted and clustered. Finally, the identified orchard information is transformed into an intuitive semantic information form, thereby obtaining a three-channel feature map. Iterative Hough linear transformation is used to detect tree rows and construct high-dimensional semantic information, including visualization graphs. Each step of the semantic mapping is visualized to verify the results. In experiments on an orchard, comparing the TEB-planner with the constructed visibility map demonstrates the superiority of our proposed navigation framework in terms of global path planning time. The global path visualization results further verify the more reasonable paths generated by the navigation planning method proposed through visibility graphs. Corresponding solutions were also proposed for the robot’s steering problem in the orchard, so that the robot can smoothly carry out a series of path planning.

Although our single-stage perceptual and semantic mapping framework has proven efficient and accurate in an orchard environment, it still suffers from its inherent limitations. Although our proposed method successfully reduced the processing time to 0.1681 s, further exploration is needed to improve the efficiency of the feature extraction. Future research should prioritize the development of a more adaptable and robust framework that can be effectively performed in different orchard scenarios. While our visibility map outperforms the TEB-planner, continued refinement and adaptation of the framework to different orchard conditions and operational needs is critical to address potential limitations. Further research should extend the proposed semantic perception and mapping method to different agricultural environments, such as litchi orchards and orange orchards, to verify its applicability and generalization.

## 6. Conclusions

This study proposes a new mapping and navigation method for robot autonomy in gardening scenarios, and details the application of autonomous robots in orchards through a semantic mapping network and navigation framework. Compared to several other mainstream methods, the semantic information extraction method proposed in this article improves detection accuracy by extracting high-dimensional semantic information, such as spatial distribution of fruit trees, tree row details, and traffic areas, combined with efficient feature extraction, network structure, and efficiency. Later, the navigation framework proposed in this article was tested in an irregular apple orchard scene. By comparing it with the TEB-planner algorithm and its improved algorithm, its effectiveness was evaluated using indicators such as planning response time. The results show that the semantic mapping navigation framework can effectively improve the response time of the robot during autonomous navigation and improve the efficiency of the robot’s path planning in the orchard.

## Figures and Tables

**Table 1:** NDT algorithm relocation results.

Location	Success Count	Average Accuracy (cm)
A	10	8.8
B	9	10.2
C	10	10.5
D	9	9.6
E	7	12.3

**Table 2:** Feature extraction strategy.

Strategy	Feature Map Time (s)	Prediction Time (s)	MIoU
vfh	2.13	0.0592	0.824
pointnet++	\	0.517	0.798
Proposed method	0.1681	0.0268	0.812

**Table 3:** Comparison of the proposed visibility graph and TEB-planner global path planning.

Scene	Planner	Average Time (ms)
Apple orchard	TEB-planner	34
Apple orchard	Visibility graph	4

## Data Availability

The data presented in this study are available on request from the corresponding author.
